# IFN-γ, IL-17 and TGF-β involvement in shaping the tumor microenvironment: The significance of modulating such cytokines in treating malignant solid tumors

**DOI:** 10.1186/1475-2867-11-33

**Published:** 2011-09-23

**Authors:** Heba A Alshaker, Khalid Z Matalka

**Affiliations:** 1 Department of Pharmacology and Biomedical Sciences, Faculty of Pharmacy and Medical Sciences, Petra University, Amman, Jordan

**Keywords:** cytokines, transcription factors, crosstalk, tumor microenvironment

## Abstract

Multiple innate and adaptive immune effector cells and molecules partake in the recognition and destruction of cancer cells to protect against growing tumors, a concept that is known as cancer immunosurveillance. Unfortunately, cancer cells are capable of avoiding this process by immunoselection of poorly immunogenic tumor cells variants along with subversion of the immune system and thus shaping both the tumor and its microenvironment. Cytokines represent part of the complex pattern of the immune response which can assist the development of cancer as well as to eliminate it. Simultaneously, a large number of cytokines may be involved in the complex interactions between host and tumor cells where this dynamic cross-talk, between tumors and the immune system, can either regulate tumor growth or tumor growth, invasion and metastasis take place. In this review, we are stressing on the interface between infiltrated immune cells and tumor cells with the emphasis on the bidirectional activities of specific cytokines: IFN-γ, TGF-β and IL-17 within the tumor microenvironment and their role in shaping it. In addition, the significance of modulating such cytokines in favor of anti-tumor response is discussed and merits the use of mixture of targeted modulators to overcome the network complexity of cytokines in the tumor microenvironment.

## 1. Introduction

The failure of the immune system to recognize and eradicate cancer cells may partly be a result of insufficient immunological activation. It is now increasingly recognized that the microenvironment plays a critical role in the progression of tumors where immune-resistant tumor variants are selected initiating the process of cancer immunoediting. Tumor-derived soluble factors can impel various mechanisms for escape from immune attack in the tumor microenvironment [1,2]. The tumor microenvironment is a pivotal factor in the course of carcinogenesis and is largely dependent on its interactions with microenvironmental components in a bidirectional way and consequently tumor progression or regression [3,4].

The tumor microenvironment was lately recognized as the product of a developing crosstalk between different cells types. In addition to tumor cells, the tumor microenvironment is comprised of immune cells, fibroblasts, stromal cells and the extracellular matrix [3]. Normal cellular microenvironment can inhibit tumor cell proliferation and cancer formation [4]. Contrariwise, as tissue becomes cancerous pathological interactions between cancer cells and host immune cells in the tumor microenvironment and lymphoid organs create an immunosuppressive network that protects the tumor from immune attack leading to tumor growth, progression as well as invasion and metastasis which is basically due to a deranged relationship between tumor and stromal cells [3-5]. In this review, we are stressing on the interface between infiltrated immune cells and tumor cells with the emphasis on the bidirectional activities of cytokines mainly IFN-γ, TGF-β and IL-17 within the tumor microenvironment and their role in shaping it. The aim, however, is to stress on the beneficial role of modulating such cytokines that favor anti-tumor activity and ultimately leads to eradicating solid tumors.

## 2. Immune cells action within tumor microenvironment

As surveillance cells in the tumor microenvironment, dendritic cells (DCs), natural killer (NK), and natural killer T cells (NKT) have been shown to infiltrate tumors and monitor the presence of novel antigen derived from tumors [6]. DCs may be activated by danger signals released from stressed or necrotic tumor cells which may include cytokines, heat shock proteins, intracellular nucleotides, and intact double-stranded DNA [6-8]. Activation and migration of DCs from the tumor site of antigen capture to secondary lymphoid organs is crucial in the initiation and amplification of immune response thereby, triggering a maturation program that includes expression of multiple costimulatory molecules and cytokines that result in efficient priming of effector T cell responses and beneficial antitumor immunity [7]. Remarkably, the cytokines produced in the local microenvironment modulate the type of response that will be generated. Conversely, DCs at the tumor microenvironment, which possess an immature phenotype, is not necessarily conducive to the activation of antitumor immune responses due to tumor-derived local immunosuppressive cytokines milieu as transforming growth factor (TGF-β), and IL-10 and growth factors as vascular endothelial growth factor (VEGF) that may instead suppress or re-conditioning DCs function and/or myeloid-derived suppressor cells (MDSCs) [7,9]. The latter would result in secreting more of TGF-β to induce dampening of T cells and the augmented regulatory T (Treg) cell function, and ultimately inducing anergy thus favoring tumor evasion. Inevitably, efficient maturation of DCs at the tumor microenvironment is pivotal for priming T cells responses and mounting effective antitumor immunity [7,10-12].

MDSCs are heterogeneous population of myeloid derived cells of immature in nature. MDSCs have the ability to suppress T cell responses, cytokine production, and promote tumor angiogenesis and metastasis [9,11]. Normally these myeloid-derived cells are located in the bone marrow differentiate to mature granulocyte, monocytes/macrophages, or dendritic cells, and only 0.5% of peripheral blood mononuclear cells remains immature. In cancer condition, however, their differentiation step is blocked, proliferate and expand into MDSCs leading to a negative impact on the immune system as a whole and more specifically in the tumor microenvironment. MDSCs expand systemically in mice (CD11b+GR1+) with transplantable tumors or spontaneous tumors and in the peripheral blood of patients (CD11+CD14-CD33+) with different types of cancer. The induction and expansion of MDSCs is initiated by factors and cytokines produced by tumor stromal cells and activated T cells [9,11]. These factors and cytokines that induce MDSCs are usually driven by chronic inflammation. For instance, prostaglandin E2, cyclooxygenase 2, IL-6, colony stimulating factor and VEGF were found to induce the differentiation of CD11b+GR1+ MDSCs from bone marrow stem cells of mice, whereas COX-2 inhibitors or IL-1 deficient mice delayed tumor progression suggesting partial mediation of MDSCs by PGE2 and/or IL-1 [9,11,13]. These studies suggest that inflammation promotes tumor progression through the induction of MDSCs that block immune surveillance and anti-tumor activity. MDSCs can directly affect T cell functions via cell-cell contact i.e. MHC-restricted and antigen-specific as well as indirectly through by suppressing CD4+ and CD8+ T cells, and inducing Treg cells [9,13]. However the latter two mechanisms depends on the subpopulation of MDSCs infiltrated in the tumor microenvironment with their produced cytokines (e.g. IL-10, TGF-β) or factors (e.g. nitric oxide, arginase, proxynitrate) [9-13].

In the course of tumor mass, infiltration of NK cells into human neoplasm's appears to correlates with a better prognosis whereas low numbers of NK cells in advanced human neoplasms indicate that NK cells do not normally home efficiently to malignant tissues [14]. The crosstalk between NK cells, DCs, and T cells initiates and sustains immune responses against tumors [15]. In this process NK cells provide early and important sources of IFN-γ production as they might be the first to recognize developing tumors and thus producing the initial levels of IFN-γ [16]. IFN-γ secreted at the tumor site by NK cells and NKT (type 1; invariant NKT) augments MHC expression on tumor cells increasing their immunogenicity which in turn can induce tumor cell death [1]. NK cells not only operate in early stage of tumor development but also act as a helper in priming process of CD8^+ ^and Th1 cells by producing IFN-γ. Likewise, NK, NKT (type 1 NKT), and γδT cells are regarded as major sources of IFN-γ during the early phase of tumor development, whereas CD4^+ ^and CD8^+ ^T cells may become additional sources as adaptive immunity evolves [17]. However, type II NKT cells were found to promote tumor progression by producing IL-13 which induces MDSCs accumulation and blocks their differentiation [18].

Growing body of evidence indicates that tumor-specific CD4^+ ^T cells and there subtypes (Th1, Th2, Treg or Th17) are directly involved in mediating *in vivo *tumor regression or evasion, as well as CD8+ T cells [19] (Table 1). Each of the latter cell subtype is regulated by a different signal transducer and activator of transcription (STAT) and transcription factor and secretes unique repertoires of cytokines that mediate their responses as well as they possess a reciprocal relation between them in normal, physiological, and diseased conditions (Table 1). In addition, naïve T helper cells (Th0) also were found to be a major cell of tumor infiltrating lymphocytes (TIL) [20]. These Th0 cells may produce IL-2, IFN-γ as well as IL-4 and may evolve in the tumor microenvironment depending on the danger signals provided in such environment [20,21]. The latter observation and the fact that cross regulation of Th1 and Th2 subsets occur through cytokine network has also been observed in TIL and peripheral blood of patients with other tumors [22]. Proportions of Th1 cells identified on the basis of intracellular production of IFN-γ are markedly reduced, whereas proportions of Th2 cells producing IL-4 are significantly elevated [23,24]. Likewise, T cells infiltrating human cervical carcinomas exhibit enhanced Th2 cytokine profiles, particularly increased IL-4 and decreased IFN-γ production [25]. Th2 polarization is dependent upon, and leads to, production of IL-4 which might have direct immunosuppressive effects on CD8^+ ^T cells at the tumor site. Furthermore, favoring Th2 development promotes tumor immune evasion leading to detrimental antitumor response [26,27]. Meanwhile, STAT1-deficient mice displayed an increase in Th2 polarization due to the block in IFN-γ signaling, and were more susceptible to tumor development [28]. These results indicate the importance of polarizing TIL to Th1 cells to control and induce regression of solid tumor.

**Table 1:** CD4^+ ^T lymphocytes and their functions in relation to transcription factors, priming and secreted cytokines in tumor progression or regression

	Th1 cells	Th2 cells	Th17 cells	CD4^+^CD25^+ ^Treg
**Priming cytokines**	IL-12 IFN-γ	IL-4	TGF-β and inflammatory cytokine (IL-1, IL-21, IL-6, TNF-α and IL-23)	TGF-β1

**Transcription factors**	T-bet STAT1	GATA3 STAT6	RORγt RORα STAT3	Foxp3 SMAD

**Major secreted effector cytokines**	IL-2 IFN-γ TNF-α	IL-4 IL-5 IL-13	IL-17A (IL-17) IL-17F IL-21 IL-22	TGF-β1 IL-10

**Role in tumor microenvironment**	Help CD8^+ ^T cells	Suppress CD8^+ ^T cells	Recruit Th1 effector T cells/Suppress CD8^+ ^T cells and promote early growth in the inflammatory environment?	Suppress CD4^+ ^and CD8^+ ^T cells

**Outcome**	Tumor regression	Tumor progression	Tumor regression/progression?	Tumor progression

Currently, Th17 cells have been shown to play important roles in inflammation and autoimmune diseases, but their exact roles in tumor immunity are still meager and contradictory [29,30]. Some studies, however, detected low number of Th17 cells in several types of tumor such as ovarian cancer, [31], non-Hodgkin's lymphoma [32], and HER2 positive breast cancer patients [33]. For instance, the levels of tumor-infiltrating Th17 cells were reduced in a group of ovarian cancer patients with more advanced disease and seemed to positively anticipate the outcome [31]. Th17 cells were able to contribute to protective human tumor immunity through recruiting effector cells to the tumor microenvironment [34]. Furthermore, tumor-specific Th17-polarized cells can eradicate large established melanoma in mice and were inversely correlated with Gleason score in prostate cancer patients [34,35]. A possible explanation lies in that Th17 CD4^+ ^T cells can be unstable and may evolve into IFN-γ-producing Th1-like cells capable of promoting tumor destruction. This effect also seems to be contingent on IFN-γ, as blocking antibodies prevented Th17 cell transfer from causing tumor regression [34].

Since Th17 cells are considered potent inducers of autoimmunity through the promotion of tissue inflammation and the mobilization of the innate immune system [19], the resulting inflammatory mediators may contribute to tumor progression by upregulating immune suppressive cells of the adaptive and innate immune systems. Substantial evidence indicates that the inflammatory reaction at a tumor site can promote tumor growth and progression [36,37]. Tumor-associated inflammatory cytokines such as IL-6 and tumor necrosis factor (TNF)-α probably regulate Th17 cells in the tumor microenvironment of ovarian cancer mouse model [38]. Also, tumor-activated monocytes (TAM) promote expansion of Th17 cells through secreting a set of key proinflammatory cytokines, such as IL-1, in the peritumoral stroma of hepatocellular carcinoma tissues [39] and ovarian cancer patients [31]. Notably, Kryczek *et al*. reported the prevalence of Th17 cells in peripheral blood and tumor microenvironment in both human and mice [40]. Elevated proportion of Th17 cells were also detected both in the peripheral blood and in the tumor-draining lymph nodes of patients with gastric cancer, which were associated with clinical stage [41]. Th17 cells were also suggested as a prognostic marker in hepatocellular carcinoma [42] suggesting that Th17 cells can also play an active role in tumor pathogenesis. Nonetheless, whether Th17 cells play the same roles in the different types and stages of cancers remains to be determined [29,30].

As a regulatory cell, CD4^+^CD25^+ ^Treg cells have emerged as being particularly critical for the maintenance of immunologic tolerance [43]. The cross-talk between Treg cells and targeted cells, such as APCs and T cells, is crucial for ensuring suppression by Treg cells in the appropriate microenvironment by soluble factors and direct cell-cell contact [44]. For instance, Treg cells inhibit DCs function through binding of CTLA-4 to CD80/86 [43-46] and IL-12-Th1 cell activation by producing TGF-β and IL-10 [45,46]. Notably, elevated proportions of CD4^+^CD25^+ ^Treg in the total CD4^+ ^T cell populations are present in the tumor microenvironment of various types of cancer where they mediate immune suppression via down regulating the functions of CD4+, CD8+, NK and NKT cells [47-49]. For instance, CD4^+^CD25^+ ^Treg cells enhance susceptibility to 3-methylcholanthrene (MCA)-induced-tumorigenesis [48] and depletion of CD4^+^CD25^+ ^Treg cells reduced tumor growth of MCA-induced fibrosarcomas [49].

Despite the disparate functions of Treg and Th17 subsets, both have been shown to be dependent on TGF-β for their differentiation [50]. Therein, TGF-β induces the Treg specific transcription factor Foxp3. However, addition of IL-6 to TGF-β enhances differentiation of Treg cells into Treg/Th17 cells [29,50]. Thus, TGF-β in the absence of inflammatory cytokines will induce Foxp3^+ ^Treg cells differentiation [50-52]. In addition, Treg/Th17 cells under the influence of IL-21 and IL-6 can differentiate into Th17 [29]. Moreover, the cytokines that promote Th17 responses significantly counteract the activation and functionality of the Tregs [29,51,52]. Thus, the cross-talk between immune cells within the tumor microenvironment involves interactions between expressed receptors on cells, types of cytokines and chemokines produced which are dependent on the stimulus or stimuli present in such environment. Such cross-talk leads to the outcome of tumor occurrence or no-tumor promotion. Therefore modulating such interactions and cytokines would be a target therapeutic tool to enhance the immune response against the tumor cells that results in eradicating them.

## 3. TGF-β, IL-17 and IFN-γ expression and activities within the tumor microenvironment

### 3.1 IFN-γ

IFN-γ is the signature cytokine produced by Th1 cells but is also derived from CD8^+ ^T cells, γδ T cells, NKT, and NK cells [16,17]. IFN-γ exerts its biologic effects by interacting with an IFN-γ receptor (IFNGR) 1 and IFNGR2 [53] resulting in activating Janus tyrosine kinase (JAK)-STAT signaling pathway which ultimately leads to phosphorylation of two STAT1 molecules followed by their dimerization and nuclear translocation [54].

IFN-γ is a cytokine that is well recognized to play a central role in coordinating tumor immune responses (Table 2). Although, few studies associated IFN-γ with tumor development via MDSCs activation and Th17 associated inflammation [55], plenty of studies have been conducted to dissect the mechanism by which IFN-γ mediates tumor rejection [56]. For instance, mice lacking IFN-γ receptors or STAT1 deficient mice developed tumors more rapidly and with higher frequency than wild type mice following a challenge with MCA [57]. An unequivocal demonstration of cancer immune surveillance is that tumor suppressor function of the immune system is critically dependent on the actions of IFN-γ which along with lymphocytes collaborate to protect against development of carcinogen-induced sarcomas and spontaneous epithelial carcinomas [56-58]. In tumor cells, IFN-γ upregulates the expression of the MHC class I antigen-processing and -presentation pathway, thereby enhancing tumor cell immunogenicity and facilitating tumor recognition and elimination by lymphocytes [58-60]. Actually, IFN-γ displays an essential role for tumor responsiveness by regulating the migration of T cells into tumor tissue [28,60]. The IFN-γ produced by tumor-infiltrating T cells might play two distinct roles in antitumor activity: activation of antitumor T cells and direct tumoricidal activity by generating inducible nitric oxide synthetase [57-61].

**Table 2:** IFN-γ, IL-17 and TGF-β effects in the tumor microenvironment

Cytokine	Secreting cells	Cytokine effects
**IFN-γ**	CD4^+ ^Th1 CD8^+ ^T NK NKT γδ T cells	**Positive effects on tumor immune surveillance:** Directs anti-proliferative and cytotoxic effects Upregulates MHC expression on tumor cells Inhibits angiogenesis Antagonizes suppression by tumor-derived TGF-β

**IL-17**	Th17 γδ T cells	**Positive and negative effects on tumor immune surveillance, depending on tumor context and model:** Suppresses tumor progression by enhancing antitumor immunity, or promote tumor progression by an increase in inflammatory angiogenesis?

**TGF-β1**	CD4^+^CD25^+^ Foxp3^+^ Treg Th17	**Negative effects on tumor immune surveillance:** Remodeling of tumor matrix and stromal cells Promotes angiogenesis Induction of Treg cells development Inhibits development, proliferation, and function of both the innate and the adaptive immunity

Studies of the benefaction of IL-12 to antitumor immunity provide further insight into the physiologically relevant stimuli for IFN-γ production to enhance antitumor immunity [61]. Cytokines as IL-12 can stimulate IFN-γ production from TH1 cells, γδ T cells, NK cells, NKT cells [16,17,62-64]. IFN-γ produced by IL-12-activated tumor-infiltrating CD8^+^T cells directly induced apoptosis of mouse hepatocellular carcinoma cells [65,66]. In addition, IFN-γ has a profound impact on solid tumors growth and metastasis and appeared to play an early role in protection from metastasis [67,68]. Developing solid tumors demand new blood vessel creation in order to grow. Partially, this occurs through both: induced outgrowth of the preexisting vasculature and de novo recruitment of vascular cell precursors from the circulation [69]. Th1 cells can impair tumor angiogenesis either directly by inhibiting endothelial cell proliferation through IFN-γ or indirectly through induction of antiangiogenic chemokines such as chemokine (C-X-C motif) ligand (CXCL)9 and CXCL10 leading to both angiostasis in and around the tumor and chemoattraction of immune effector cells into the tumor site [61,70]. Moreover, IFN-γ can antagonize the production of immunosuppressive cytokines such as TGF-β and IL-10 which can promote the development of otherwise highly immunogenic tumors, improves the establishment of an effective antitumor memory immune response, and thus controls ongoing tumor growth [66]. However, recent studies have shown that Eriobotrya japonica hydrophilic extract (EJHE) induced IL-12, IFN-γ and TNF-α *in vivo *and *in vitro *more than IL-10 [71], and increased IFN-γ levels, decreased TGF-β levels but did not change IL-17 in spleen of normal mice [72]. Even though continuous administration of EHJE increased IFN-γ levels in the tumor microenvironment as well as TGF-β and IL-17, the survival of Meth-A fibrosarcoma bearing mice was significantly increased [72]. This indicates the complexity of the tumor-infiltrated immune cells such as MDSCs, Treg, and TH17 as well as their differentiation stages, immune-cytokine network and the type of tumor and its sensitivity to anti-tumor/pro-tumor cytokines.

### 3.2 IL-17

IL-17 or (IL-17A) is the original member of IL-17 family which further includes IL17B, IL17C, IL17D, IL17E (also called IL-25), and IL17F which displays the highest degree of homology with IL-17A [73,74]. IL-17 is the signature cytokine of a unique lineage of CD4^+ ^T cells termed Th17 [73]. Additional cellular sources of IL-17A include γδT cells, CD8^+ ^T cells, neutrophils and eosinophils [73,74]. IL-17 is also produced by NKT cells upon TCR stimulation [74]. IL-17 binds to and signals through IL-17 receptor A (IL-17RA) which is widely expressed on epithelial cells, endothelial cells and fibroblasts. The ligation of IL-17/IL-17R results in the release of inflammatory mediators like IL-1, IL-6, IL-8, IL-23, TNF-α and several chemokines to further stimulate the inflammatory cascade. Since inflammation is regarded as a promoter to carcinogenesis [36], evaluating IL-17 in cancer development is a necessity.

IL-17 secreting cells have been identified in many types of human cancers and in murine models as well, however, there are discrepant data on a possible role for IL-17 in carcinogenesis as its precise role is still comparatively poorly understood [29,30]. A series of reports have suggested potent antitumor functions for IL-17 and IL-17 producing T cell subsets [29-35,75-77]. Antitumor activity of IL-17 could be achieved by means of a T cell-dependent mechanism as in an increased generation of specific CTLs [75]. Transfection of IL-17 into Meth-A fibrosarcoma cell lines was shown to augment the expression of both MHC class I and II antigens, therefore inducing tumor specific antitumor immunity [76]. Tumor growth and lung metastasis were intensified in IL-17 deficient mice whereas *in vitro *TGF-β and IL-6 polarized Th17 cells induced tumor regression [77]. Similarly, IL-17 deficient mice were more susceptible to developing lung melanoma than wild-type mice, a phenotype that was blocked by adoptive transfer of tumor specific Th17 cells [78]. Transferred Th17 cells prevented tumor development and exhibited stronger therapeutic efficacy, than Th1 cells, fostered immune surveillance by CD8^+ ^CTLs and DCs implying that the stimulation of IFN-γ production by IL-17 is needed for IL-17 exerted antitumor response in IL-17 deficient mice [78].

Notwithstanding, other reports have suggested potent protumor functions for IL-17 and IL-17 producing T cell subsets. Whilst IFN-γ disrupts developing tumor's vasculature [60], it is established that IL-17 is as an angiogenic factor that act on endothelial, stromal and tumor cells. IL-17 stimulates the migration of vascular endothelial cells *in vitro *and elicits vessel formation *in vivo *to induce tumor vascularization and promote tumor growth [79-81]. Coincidently, defects in IFN-γR are associated with increased tumor growth whereas IL-17R-deficient mice display reduced tumor development. Blockade of IL-17 even reverses the susceptibility of IFN-γR^-/- ^mice to tumor development indicating that IL-17-mediated responses at tumor sites promote tumor development [81]. Additionally, the latter report also showed that a defect in IL-17R reduces IFN-γ production by T cells and that tumor growth is inhibited in IL-17/IFN-γR double-knockout mice suggesting that IFN-γ appears to play a minor role in IL-17-mediated regulation of tumor development [82]. This observation is also confirmed by a previous report showing inhibition of tumor development in IL-17/IFN-γ double-knockout mice [83]. It has to be mentioned also that IL-17R deficiency increased CD8 T cells and reduced MDSCs tumor infiltration [82], and systemic administration of IL-17 promoted tumor growth, enhanced MDSCs and reduced CD8 T cells tumor infiltration [82]. Furthermore, it has been shown recently in a spontaneous model of intestinal tumorigenesis (driven by a heterozygote mutation in the tumor suppressor gene denomatous polyposis coli) that the ablation of IL-17 leads to inhibition of tumor development [84]. Additionally, IL-17 promoted the growth rate and tumorigenicity of human cervical tumors transplanted into athymic nude mice [85]; knockdown of the IL-17 receptor in 4T1 mouse mammary cancer cells decreased tumor growth *in vivo *[86]; and IL-17 depletion delays development of chemically induced papillomas [55]. Remarkably, these effects seem to be driven by a pathway of IL-17 inducing IL-6 production by both neoplastic and stromal cells, which successively leads to activation of STAT3 [83]. Such activation of STAT3 in tumor cells and tumor-associated inflammatory cells plays a critical role in tumor progression by augmenting tumor survival and tumor angiogenesis, and suppressing antitumor immunity [87]. Seemingly, not merely STAT3 is involved but also a number of inflammatory cytokines does play critical roles in this process and many reports indicate that IL-17 in the context of inflammations exerts tumor promoting effects [38,87].

Proinflammatory cytokine as IL-23 is an important cytokine for the expansion and survival of Th17 cells [88]. IL-23 demonstrated to be an important link between tumor-promoting and proinflammatory processes. IL-23p19 mRNA was upregulated in significant manner in the majority of cancer samples from various organ types, comprising colon, ovarian, lung, breast, and stomach cancers as well as melanoma [88]. STAT3 signaling within the tumor microenvironment induces the protumor cytokine, IL-23, while inhibiting a central antitumor cytokine, IL-12, thereby shifting the balance of tumor immunity toward carcinogenesis [88,89]. In the course of the tumor microenvironment, TNF-α enhanced tumor growth via the inflammatory cytokine IL-17 in a mouse model of ovarian cancer and in patients with advanced cancer [38]. Moreover, a set of key cytokines profile (IL-1β, IL-6, TNF-α, and TGF-β) was detected in ovarian tumor cells, tumor-derived fibroblasts, and APCs, which formed a cytokine milieu that regulated and expanded human IL-17-producing Th17 cells [90] suggesting that these inflammatory cytokines within the tumor microenvironment can provide a favorable niche for developing the unstable Th17 cells and potentially directing them into a dangerous feedback loop.

### 3.3 TGF-β1

TGF-β is a member of a large family of developmental conserved proteins. Three closely related isoforms have been identified of this family including TGF-β1, TGF-β2 and TGF-β3 [91]. TGF-β1 is the predominant isoform expressed in the immune system and lymphoid organs and is essential for maintaining T cell homeostasis, Treg cells and effector cells function and carcinogenesis [91]. TGF-β binds type I and II TGF-β receptors causing phosphorylation of the downstream mediators SMAD 2 and 3. The phosphorylated SMAD2 and 3 combine to SMAD4 and enter the nucleus to modulate gene expression.

TGF-β signaling is contextual, depends on the cell type, and has both positive and negative effects on cancer. Specifically, in cancer TGF-β exerts a perplexed role. Initially, it acts as a tumor suppressor since it induces apoptosis and inhibits the growth of cells [91,92]. In mice, transgenic expression of TGF-β1 in mammary gland promoted growth arrest and in some settings enhanced apoptosis [91]. However, changes in TGF-β signaling often correlate with tumor stage and rate of progression [91,92]. At later stages of tumor progression, TGF-β acts as a tumor promoter. Seemingly at this stage cancer cells protect themselves and tend to acquire increasing resistance to ignore TGF-β growth inhibitory signals which is an important reason for the shift from being a tumor suppressor to a tumor promoter. Subsequently, cancer cells start secreting non-physiological levels of TGF-β in an autocrine and paracrine manner which may affect the differentiation of the tumor cells and the surrounding cellular environment, respectively, leading to development of the tumor and metastasis in an immunosuppressive environment that is rich in TGF-β [93]. Deletion of the type II TGF-β receptor gene (*Tgfbr2*) in mammary carcinomas results in the recruitment of Gr-1^+^CD11b^+ ^MDSCs that directly promoted tumor metastasis through enhanced metalloproteinase and TGF-β production [94].

In the context of malignant niche, TGF-β induces epithelial transition that in turn converts cancer cells into invasive cells. Tumor-derived TGF-β causes remodeling of the tumor matrix: by acting on stromal fibroblasts; inducing the expression of mitogenic signals towards the carcinoma cells; and by promoting angiogenesis through stimulating VEGF and other angiogenic factors production which correlates to increased invasiveness and metastasis [91-95]. Additionally, suppression of the immune system by TGF-β also contributes to its tumor promoting effects. In the perspective of the complex interplay between the immune system and tumor microenvironment, TGF-β is an immunosuppressive cytokine that function at several levels in the tumor microenvironment which also encompasses reduction in the amount of antigen presentation by DCs [12-14], reduction in the proliferation of T cells (Th1, Th2 and CTL), suppression of NK cells cytotoxic activity, and stimulation of Treg cells proliferation thus impeding immune surveillance of the developing tumor [91,92]. Nam and his colleagues (2008) have shown that presence of TGF-β in a tumor bed contributes to a local cytokine milieu that can subvert CD8^+ ^T cell differentiation or expansion down a proinflammatory, IL-17-secreting path [86]. Moreover, knockdown of the IL-17 receptor in 4T1 mouse mammary cancer cells enhanced apoptosis and decreased tumor growth *in vivo *confirming that tumor cells evolved to use IL-17 as a survival factor as one of the consequences of TGF-β overexpression [89].

Being one of the most commonly used drugs in cancer chemotherapy, Doxorubicin, is a DNA-damaging drug that possesses strong antineoplastic activity. Doxorubicin specifically inhibited TGF-β-signaling in human lung adenocarcinoma A549 cells by blocking TGF-β1-induced activation of SMAD3-responsive CAGA_12_-Luc reporter while other drugs like Cisplatin or Methotrexate did not alter activation of CAGA_12_-Luc reporter under the same conditions suggesting that that such inhibitory effect could be a novel mechanism of Doxorubicin action towards tumor cells [96]. Measurement of IFN-γ and IL-17 were not reported in the latter work, however, molecular targeting of TGF-β production or disruption of the TGF-β pathway could not only improve host immunosurveillance but also inhibit tumor progression by upregulation of IFN-γ, and provide additional therapeutic options through manipulation of IL-17 levels. Moreover, strong evidence suggests that therapeutic intervention in TGF-β signalling via small molecule, antibody or antisense TGF-β antagonists are needed to fulfill one important aspect of treating solid malignant tumors [92,97].

## 4. The paradigm role of TGF-β, IL-17 and IFN-γ within tumor microenvironment

Since the circular nature of the relationship between inflammation and cancer has proven to be evident, it is important to emphasize the following generalizations; inflammation can cause cancer; inflammation can cause mutation; mutation can cause inflammation; mutation can cause cancer; cancer can cause inflammation; and inflammation can suppress cancer [98]. In addition, inflammation can modulate the immune response to enhance tumor growth [17,21,26,40]. Since TGF-β is conventionally regarded as an anti-inflammatory cytokine whereas IFN-γ and IL-17 are considered proinflammatory cytokines, the paradigm role of these cytokines is complex in cancer immunology and tumor microenvironment. TGF-β demonstrates both tumor suppressor and oncogenic activities [92]. In the current paradigm, the suppressor activities dominate in normal tissue. On the other hand, changes in TGF-β expression and cellular responses during tumorigenesis tip the balance in favor of its oncogenic activities to drive malignant progression, invasion and metastasis both *in vitro *and *in vivo *[92,99]. In fact, understanding the roles of TGF-β produced from T cells, Treg, MDSCs and even B regulatory cells within the tumor microenvironment requires insight into the changing response patterns of many interacting cell types [11,92,100,101]. For instance, mutations in type II TGF-β receptor gene, *TGFBR2*, are frequent in human colon cancer with microsatellite instability [102], and down regulation of TGF-β-SMAD signaling pathway is associated with poor prognosis in breast cancer patients [103]. Furthermore, deletion of *TGFBR2*, in mammary epithelial cells results in increasing chemokines that recruit further MDSCs [94]. These MDSCs secretes high levels of TGF-β and metalloproteinases [94]. In a tumor microenvironment, the latter two enhances tumor growth and metastasis. In addition, loss of *TGFBR2 *in fibroblasts or T cells leads to prostate intraepithelial neoplasia and colon carcinoma [104]. These data suggest controlling inflammation reduces tumorigenesis and inflammation in an established tumor enhances it growth and metastasis. On the other hand, TGF-β, as a tumor promoter and considered to be pre-oncogenic, is produced from many tumor types. For instance, TGF-β-SMAD activity is usually high in aggressive gliomas [105]. Several studies showed the blocking TGF-β reduced tumor progression through several parameters including high infiltration of CD8+ T cells and suppression of Treg and MDSCs [93,86,106].

Th17 cells differentiation requires TGF-β in humans and mice as well. However, TGF-β alone is not sufficient for the induction of Th17 in mice as it requires IL-6 plus TGF-β [77,83,107,108]. Actually, the discovery that TGF-β is a crucial cytokine for Th17 cell development suggested that Th17 and Treg cell subsets share reciprocal developmental pathways from uncommitted CD4^+ ^precursors [50]. Moreover, there is a direct lineage relationship of Th17 cells with Treg cells. Indeed, these two subsets require TGF-β for lineage commitment and it is further observed that there are direct interactions between the lineage specific transcription factor Foxp3 of Treg and RORγt of Th17. Further work showed that TGF-β signaled in a concentration dependent manner to promote the expression of both Foxp3 and RORγt. Foxp3 directly bound to RORγt preventing Th17 differentiation; an effect relieved by IL-6, IL-21 and IL-23, therefore confirming the suppressive function of Foxp3 on RORγt [52]. Practically, such interactions can't be disregarded within local tumor microenvironment but even still need to be fully further investigated. A study has revealed that in parallel to high levels of CD4^+^Foxp3^+ ^Treg cells, IL-17^+ ^T cells including CD4^+ ^T cells and CD8^+ ^T cells were kinetically induced in the tumor microenvironment in multiple mouse and human tumors. Furthermore, IL-17^+ ^T cells demonstrated a dynamic differentiation; Th17/Treg, Th17, and Th17/Th1 in the tumor microenvironment thus providing new insight of IL-17^+ ^T cells potential role in tumor immune pathology and therapy [40].

Concerning tumor development, some human tumor cells could express IL-17 that represents an early event in the development of the inflammatory reaction within the tumor microenvironment which may successively influence tumor phenotype and growth [29]. Ostensibly, IL-17 seems to be a pleiotropic cytokine, as other inflammatory cytokines, with possible protumor or antitumor effects which often depends on the immunogenicity and degree of inflammation in the tumor itself [29,82]. Meth-A cells transfected with the human IL-17 gene can induce tumor-specific antitumor immunity by augmenting the expression of major histocompatibility complex (MHC) class I and II antigens; this antitumor immunity may be mediated by CD4^+ ^and CD8^+ ^T cells [76]. On the other hand, using IL-17 knockout mice, Wang et al. showed that disruption of IL-17 reduced tumorigenesis and it was associated with less STAT3 activation, STAT3-associated proliferative and antiapoptotic gene expression, hyperplasia, and MDSCs tumor infiltration within the tumor microenvironment [83]. The latter results indicate the role of IL-17-STAT3 pathway in cancer-associated inflammation in the tumor microenvironment. Furthermore, IL-17 up-regulated elaboration of a variety of proangiogenic factors by fibroblasts as well as tumor cells revealing a novel role for IL-17 as a CD4 or γδT cell-derived mediator as a tumor promoter by inducing angiogenesis and tumor growth [80,83]. A recent work showed that G-197A allele of the *IL-17A *gene promoter was significantly associated with an increased risk of subsequent development of intestinal-type gastric cancer upon its association with the progression of gastric mucosal inflammation and the development of gastric mucosal atrophy [109]. In addition, a very recent report by Kryczek et al. [110] showed for the first time an IL-17+ regulatory T cells expressing FoxP3 (IL-17+Foxp3+CD4+ T cells) in the tumor microenvironment of inflammatory tumors such as colon cancer as well as chronic inflammation tissue of the colon but not in renal cell carcinoma, melanoma or ovarian carcinomas. The latter is supported by the two stage skin carcinogenesis model using 7,12-dimethyl benz(a) anthracene/12-O-tetradecanoylphorbel-13-acetate (TPA)-induced papilloma where the latter upregalated IL-17 expression in the skin papilloma and TH17 in the draining lymph nodes. Depletion or neutralization of IL-17 led reduced keratinocyte proliferation and delayed papilloma development [55]. However, the latter observations were also correlated with IFNγ R deficiency or neutralization of IFN-γ and IFN-γ response to induce the expression of TNF-α, IL-6, TGF-β during the papilloma promoting stage. This stresses that the differential behavior of IL-17 within inflammatory versus non-inflammatory solid tumors and opens a new strategy for suppressing these cells in inflammation-induced solid tumors via down regulating SMAD3/4 and STAT3 signaling pathways. Similarly, it was found that *in vivo *inactivation of genes that govern MDSCs accumulation, such as STAT3 and STAT6 restores T cell function and promotes tumor regression [13].

The mouse prostate cancer cell line TRAMP-C2 secretes TGF-β1 and show low MHC-I expression. Treatment with IFN-γ increased MHC-I expression and antagonized the immunosuppressant activity of TGF-β [66]. Since IFN-γ and TGF-β show reciprocal antagonistic effects, treatment of TRAMP-C2 with IFN-γ not only restored MHC-I expression but also improved the establishment of an effective antitumor memory immune response and thus the control of ongoing tumor growth [66]. *In vitro*, studies with hepatic stellate cells demonstrate that TGF-β-dependent activation of SMAD3/4 responsive reporter construct was significantly decreased by IFN-γ due to the fact that IFN-γ induced the activity of the SMAD7 promoter and SMAD7 protein expression via STAT-1 signaling providing a novel approach for opposing profibrogenic activities of TGF-β in liver cells and indicating a TGF-β antagonizing function by IFN-γ [96].

Treatment of cancer is critically dependent on IFN-γ and it is ability to activate macrophages, T cytotoxic, NK cells, and regulate MDSCS, Treg cells. However, IFN-γ relation to IL-17 and TH17 cells depending on the tumor environment context and the stability of TH17 [55,110]. Nonetheless, the finding that IFN-γ receptor knockout mice exhibit severely impaired antitumor capability illustrated the importance of IFN-γ in tumor immunity and the induction of IFN-γ via STAT1 activation and inhibition of STAT6 and 3. All in all, IFN-γ is correlated with several direct and indirect antitumor properties and tumor responsiveness to IFN-γ is necessary for IFN-γ-dependent inhibition of tumor angiogenesis by CD4^+ ^T cells.

## 5. Conclusions and future directions

It is clear that IFN-γ, IL-17 and TGF-β are capable of exerting multiple effects within tumor microenvironment site, many of the exerted effects are contextual hence, compelling further investigations in that regard along with any attempt of their modulation that should be approached with a definite knowledge of the initial level of cytokines and their complex interaction with particular type of tumors and the immune cells. Such knowledge might better be enhanced through studying the gene expression of cytokines and gene-expression signatures in relation of type of infiltrating immune cells within the tumor microenvironment.

Since in the tumor microenvironment the targeted cells react differently in context with different stimuli, care should be taken in the creation of therapeutic interventions utilizing compounds that could stable the delicately re-harmonized equilibrium that exists at the base of cytokine network. Additional evidence for the criticality of cytokines in cancer is their antagonistic efficacy as aforementioned. Therefore, we propose that modulating cytokines' effects on immune response in the tumor microenvironment are potential sources for a favorable outcome (Table 3). For instance, immunomodulators that have the capacity to induce IL-12 and IFN-γ with novel SMAD3/4 or STAT3 and STAT6 inhibitory molecules are potential targeted mixtures of polarizing the beneficial immune cells within tumor microenvironment. Furthermore, such mixtures with known chemotherapeutic agents that are potentially selective would be more potent in treating malignant solid tumors.

**Table 3:** The rationale of modulating each cytokine with the favorable outcome of each transcription factor involved.

Cytokine	Rationale	Favorable outcome
**IFN-γ**	**Tumor growth inhibition:** Stimulation of cellular immunity; Antagonizes TGF-β1 deleterious effects; Inhibits IL-17 production	↑ STAT1 activation

**IL-12**	**Tumor growth inhibition:** Acts via IFN-γ and activates NK, Th1, CD8+ T cell and promotes DCs maturation	↑ STAT4 activation

**IL-17**	**Tumor growth promotion:** TGF-β1 with an inflammatory cytokine supports IL-17 production via STAT3 activation; Inhibits IL-12 favorable effects by STAT3 activation	↓ STAT3 activation

**TGF-β1**	**Tumor growth promotion**: Inhibits IFN-γ favorable effects Supports Treg cells differentiation; Supports Th17 cells differentiation with an inflammatory cytokine	↓ SMAD3 activation

## Competing interests

The authors declare that they have no competing interests.

## Authors' contributions

HA did the literature search and drafted the manuscript. KM conceived the idea and did literature search on specific points and finalized the manuscript. Both authors approved the final version.
